# Pepsin.—Its Therapeutic Value

**Published:** 1873-11

**Authors:** T. Curtis Smith

**Affiliations:** Middleport, O.


### SOUTHERN MEDICAL RECORD.

V[illegible] III.—NOVEMBER, 1873.—[illegible]. 11.

### PART I.

### ORIGINAL COMMUNICATIONS.

### PEPSIN.—ITS THERAPEUTIC VALUE.

BY T. CURTIS SMITH, M. D., MIDDLEPORT, O.

Pepsin is one of those *old, old* agents which was used scores of years ago, and which, though keeping its name above the continual flood of new agents which are constantly rising to arrest the attention and study of the members of the medical profession, has nevertheless not yet reached the height which it deservedly should occupy as a therapeutic agent. It has been known and used so long ago that it is difficult to decide who first discovered its properties, or brought it to our notice. A hundred years ago it was no very uncommon thing for the pig's stomach, fresh, to be macerated, and the resulting fluid used for both acute and chronic digestive and bowel troubles. Yet it was not a popular remedy. Nor has it been a half decade since its use in the more refined shape—as pepsin—has begun to be popular for digestive derangements, and even now, it is not used half as often as it should be in such diseases.

Many of our older physicians don't use it, because they have become habituated to other agents. Others look upon it as a kind of innovation, and refuse to accept it in consequence. Again, others have resorted to it, and from the misfortune of giving it in too small doses, or of using an impure or deteriorated agent, have failed to secure good results. Only a few days since I heard an old and well-informed physician say he would not use pepsin, as he had no faith in its power as a digestive agent. He had not given it a trial; could get along without it, and would, in places where it should be used, prescribe mineral acids, mineral tonics, strychnine, &c., agents tenfold more liable to prove hurtful than pepsin.

Pepsin is that nitrogenized, catalytic, active principle that gives digestive power to the gastric juices, and that of commerce is usually obtained from the stomach of the calf, hog or sheep. It is not necessary here to describe the process of its manufacture. I have found that manufactured by E. Schaffe, of Louisville, Ky., to be very efficient and pleasant to take.

The habitat of this principle being in the stomach, it is natural to conclude that its presence in that organ in normal quantity would go far towards securing healthy digestion, and that a deficiency in quantity or defect in quality would be likely to secure only imperfect, or often painful digestion.

By this statement I do not wish to be understood as claiming that the absence of this agent in the stomach is always the cause of indigestion and imperfect assimilation, for it is nearly always present under all circumstances, in greater or less amount. *Per contra,* we know that many other causes may arise, producing imperfect stomachic digestion, in which case some other treatment must be adopted besides the simple resort to pepsin, which, however, even here, will often prove to be a clever aid.

Pepsin taken into the stomach produces no very well marked physiological symptoms, even if taken quite largely; and its action seems to be that of establishing an artificial digestion or of simply aiding normal digestion, which not being painful, or marked by any very distinct symptoms, it could not be expected that pepsin would cause symptoms more plainly marked if taken in health. Taken in imperfect or painful digestion, its action is often soon apparent by the relief afforded and the feeling of comfort and satiety which seems to follow its use. It should be taken just before or just after meals. If the appetite is poor, *i. e.,* little desire for food isfelt, and what is taken is, as patients often say "forced down," it is better to give it before meals. If on the other hand the appetite is ravenous, with digestion more or less imperfectly performed, or is attended with uneasy or painful sensations, it should be taken immediately after eating. In the former instance, taken a few minutes in advance, it is a good appetizer, and will assist in reducing the food to a consistence proper for absorption. In the latter case it simply aids digestion by giving better tone to the stomach and probably carries a part of the process on artificially, thus relieving the stomach of a part of its labor.

Whenever taken, it should not be followed with the use of very hot food or drink, as anything above 112 deg. Fhr., destroys more or less its digestive power. It is more effective at the natural temperature of the body.

I think I have found it most effectual administered in powder or in weak solution with hydrochloric acid. In either form the full power of the agent is developed. If given with syrup, glycerine, or any similar vehicle, it should be prepared at the time it is taken and not allowed to stand. A mixture of glycerine or syrup and pepsin soon loses its power, for the latter spends its influence in digesting artificially the vehicle with which it is so mixed. Alcoholic preparations of pepsin destroy its activity to a considerable extent. These reasons are often the cause of a loss of confidence in the beneficial influence of the agent.

To a child it may, if difficult to get to take medicine, be given in a drink of water, cold tea, coffee or milk, or on bread and butter, as it is nearly tasteless. It is one of our most harmless remedies, and is, in suitable cases, one of the most effectual.

What are its therapeutic applications? Naturally, one would suppose, from the foregoing, that it would prove the *sine qua non* in dyspepsia, and so it is in cases of simple stomachic debility, in defective quality or deficient quantity of gastric juice. It will enable persons thus affected, and who suffer uneasy or painful digestion, to take a full and hearty meal with impunity. Especially is it adapted to those cases which cannot readily digest oily or animal food. Gastralgia from indigestion readily yields to a few doses of the agent, say x to xx grs. If other morbid conditions prevail than those above named, it will then only prove to be a very useful adjuvant. It is not expected to cure structural changes of the stomach alone, but it will assist digestion and assimilation in many of those cases, thus adding support to the system and allowing a better opportunity for recovery from them by the use of other agents.

It is no uncommon occurrence to meet with patients who eat sufficently, but who are troubled with acidity of the stomach, and have occasional diarrhœa, during which the ingesta, taken hours before, is carried off in a simply emulsified state. Digestion here is only partial, and as a result of this, the food is but partially assimilated; the patient has become anemic, with more or less emaciation and general uneasy abdominal feeling, and of more or less weakness and lassitude. In such cases, the use of pepsin is very strongly indicated, and would be better given in solution with hydrochloric acid.

Very many such cases occur among children, especially during the period of dentiton, and among those artificially fed. The stomach of the young infant very often proves to be unable to digest cow's milk, and it vomits it or ejects it per rectum in a curdled state, with a very offensive odor. A little pepsin, grs. v to x, added to its food, or given immediately after it is taken, will soon change this abnormal digestion to a perfectly healthy state, and it is surprising, often, to see how the little waifs fatten under its use. It can be continued in such cases indefinitely, without the least harm resulting from its use.

Dr. A. Davidson, Asst. Physician to the Children's Infirmary, Liverpool, in the Practitioner, makes the following statement: "There is a form of diarrhœa often observed in young children, from one to two years old, (the period of dentition), in which the ordinary treatment by anticids, aromatics, astringents, &c., has no good effect; but if pepsin be administered, it scarcely ever fails to restore the child at once to health. The diarrhœa in this instance arises from feebleness of the digestive powers. A large proportion of the food is not assimilated, and consequently passes on and appears in the motions in an undigested state."

Almost daily such cases come under the observation of every physician, and they occur not simply in "hospital practice," but everywhere, especially during the hot months. Like Dr. Davidson, I have found the treatment of this kind of diarrhœa by aromatics, astringents, and antacids, as "utterly useless," and not in accordance with reason or common sense.

In accordance with the above, Dr. W. J. Cummins, in the Dub. Jour. Med. Sci., Feb., 1872, gives his views, warmly advocating the use of pepsin in those cases where the gastric juice in the child's stomach is unable to perform its duty thoroughly. He says, "The remedy is simple for this state of things, for although we cannot change the elementary composition of the milk we have to use, we can introduce into the infant's stomach a digestive power proportionate to the food it has to use—the organic principle of digestion taken from the stomach of the calf."

It is not expected that pepsin should be used to the exclusion of everything else, but that experience teaches that in this class of cases, it is the main reliance for speedy, permanent and harmless relief. Dr. Cummins further states that he has used this agent for many years in practice, (he prefers the wine of pepsin), during which, "in dyspensary, hospital, and private practice, I have seen many apparently hopeless cases recover under its use." Dr. J. S. Hawley, of Brooklyn, N. Y., has recently published a paper on this subject, highly commending this agent in the "diarrhœa of infants," and as extravagant as his paper may seem, it appears to me as not going a hair's breadth too far in the laudation of this simple, valuable agent.

He also mentions its value in that scourge of infancy, cholera infantum. To me, it seems that after the acute stage has passed, the stomach and alimentary canal having been well emptied, that no agent now in our possession can compare with it in value. In former years, it was my lot to witness the decease of children frequently, from this disease, after it had run a long course, death always occurring from inanition, the stomach being unable to digest or assimilate the blandest food. This I observed, not only in my own, but the practice of others. Then the aromatics, astringents and anticids were all tried in vain. True, many cases recovered, but the mortality list was too great to be looked upon with perfect placidity of mind. Latterly, under the free use of pepsin, I am well satisfied that the list is very greatly cut down, and that instead of having such cases on hand for several days—often for weeks—a very short course of treatment suffices to effect relief.

Of course it is not expected of the agent to prove perfectly satisfactory whether the exciting cause is removed or not. Common sense teaches us to look to that indispensable part of the treatment, but even when the cause is removed as far as we can, the digestive powers are often unable to regain their former normal status, and the disease goes on. Again, in children artificially fed and also where the mother's or nurse's milk is not readily digestible, the cause often cannot be removed. The child must be fed or it will starve, and circumstances very often render it impossible to secure other food than that which the child already receives. What can we do in such a case ? Simply add to the stomach a digestive power that will enable it to digest and assimilate the food it is compelled to take in order to sustain life at all. That *digestive power we have in pepsin,* as we have in no other agent. It may seem extravagant and possibly it is so, but I would rather have a sufficiency of good pepsin to treat that class of diarrhea above mentiond, and also cholera infantum, after the acute stage is passed, than all other therapeutic agents combined. Only this morning, a parent presented himself saying that he must have some more of those powders for his child, as it had another attack of diarrhea and they always checked it without making him sick or causing any trouble. This was the case of an artificially fed infant, whose mother is in the last stage of tuberculosis, and whose surroundings are far from being hygienic. Yet under these unfavorable conditions the infant thrives well by an occcsional resort to pepsin. Such cases, and many others more favorably constituted and well situated, will not only yield readily to its use, but with a fair degree of permanency.

In cholera infantum, I have in the last year, treated quite a large number almost exclusively with pepsin, with entire satisfaction. I will give but a case or two.

In the early part of June, '73, I was called to attend a little boy three years old. He had been vomiting and purging for two days quite freely; had considerable griping; pulse 135; feeble; skin pale and cool with occasional febrile exacerbations; tongue coated yellow through the middle and very red at either side: thirst excessive. I gave him cold drinks in small quantities often; applied counter irritation over the bowels; directed light diet; gave mistura creta, with glycerine and camph. tinct. opii, the first day. The contents of the bowels had already been removed by a free use of oil, by the parents.

On the second day the child seemed a little improved, and therefore the treatment was continued, and so I followed this line for several days; but the improvement was only temporary. He soon became greatly reduced, with the disease continuing. I had kept him thus far on this plan, to try to prove that the pepsin was not as valuable as I might have led myself to believe. At this stage I put him on pepsin, in v. gr. doses every three hours. After the third dose there was no more vomiting, and in a few hours the purging ceased, after which, there was no hindrance to a rapid improvement.

In August, I attended a little boy afflicted with cholera infantum. He had been sick two weeks, his maternal parent having thought herself equal to the task of curing him. At the date of my first visit he was greatly emaciated; feverish; vomiting frequently; obstinate diarrhea; everything taken seemed to be rejected per orum or per anum. I immediately placed him on large doses of pepsin, without any other treatment or any reference to directions concerning diet. The second day he was but little, if any better, the third day he was greatly improved and was soon well again.

A little case of an infant ten months old, came a month since under my observation. Its diarrhea was profuse; vomited occasionally and seemed in much pain. I gave it a mild purgative, followed that with tannin, paregoric and syrup, which failed to benefit it. I then gave it creta preparata combined with an aromatic and astringent. This accomplished no benefit. I now examined its stools, and found much of its food in them undigested. Of course I resorted at once to pepsin, and with the satisfaction of seeing the case rapidly recover, with the use of a few free doses.

With all the commendation we give this agent, we do not wish to be understood that it is not proper to use other agents in the above diseases, according to the condition in which we find each case, but simply that it is a highly useful remedy, and if well used there will be few cases run to the extreme condition which we sometimes see from cholera infantum and chronic diarrhea in children.

Another class of infantile cases we see occasionally, is that of inanition, in which the child takes but little food, and what it does digest seems not to be assimilated, or it may take a sufficient quantity or even too much food, and still continue to dwindle into a state of marasmus for the want of power to digest and assimilate the aliment taken. Here again we need an additional digestive power, which is had in the addition of pepsin to the weak gastric juice of the infant's stomach, which will prove of the greatest utility in these cases. It is equally beneficial in cases of disassimilation at all ages where it depends on want of digestive power from inefficient gastric juice.

Another trouble to which pepsin is now being frequently applied is the nausea and vomiting of pregnancy. While it is often very beneficial here, it will not invariably prove successful, but I have seldom seen a combination of pepsin and oxalate of cerium fail to do good. Using grs. xv. of the former to grs iii to v. of the latter, from one to three times a day as may be required

I know it has been claimed that pepsin will produce catharsis, (D. A. Morse, of Midway, Ohio,) but I have taken it repeatedly to learn if such effects followed its use, and have failed in every instance to secure cathartic action, even in ʒ ss doses. I have also failed to observe catharsis from its liberal use in many of my patients. Neither will it produce constipation when given in a state of health.

If my observations have been erroneous I am open to conviction on satisfactory proof of the same.

